# CNNs for automatic glaucoma assessment using fundus images: an extensive validation

**DOI:** 10.1186/s12938-019-0649-y

**Published:** 2019-03-20

**Authors:** Andres Diaz-Pinto, Sandra Morales, Valery Naranjo, Thomas Köhler, Jose M. Mossi, Amparo Navea

**Affiliations:** 1 Instituto de Investigación e Innovación en Bioingeniería, I3B, Universitat Politècnica de València, Camino de Vera s/n, 46022 Valencia, Spain; 2 Pattern Recognition Lab, University of Erlangen-Nuremberg, Erlangen, Germany; 3 iTEAM, Universitat Politècnica de València, Camino de Vera s/n, 46022 Valencia, Spain; 4 Instituto de Ciencias Biomédicas, Universidad CEU Cardenal Herrera, Avenida del Seminario s/n, Moncada, 46313 Valencia, Spain

**Keywords:** Glaucoma, ACRIMA database, Fundus images, CNN, Fine-tuning

## Abstract

**Background:**

Most current algorithms for automatic glaucoma assessment using fundus images rely on handcrafted features based on segmentation, which are affected by the performance of the chosen segmentation method and the extracted features. Among other characteristics, convolutional neural networks (CNNs) are known because of their ability to learn highly discriminative features from raw pixel intensities.

**Methods:**

In this paper, we employed five different ImageNet-trained models (VGG16, VGG19, InceptionV3, ResNet50 and Xception) for automatic glaucoma assessment using fundus images. Results from an extensive validation using cross-validation and cross-testing strategies were compared with previous works in the literature.

**Results:**

Using five public databases (1707 images), an average AUC of *0.9605* with a 95% confidence interval of 95.92–97.07%, an average specificity of *0.8580* and an average sensitivity of *0.9346* were obtained after using the Xception architecture, significantly improving the performance of other state-of-the-art works. Moreover, a new clinical database, ACRIMA, has been made publicly available, containing 705 labelled images. It is composed of 396 glaucomatous images and 309 normal images, which means, the largest public database for glaucoma diagnosis. The high specificity and sensitivity obtained from the proposed approach are supported by an extensive validation using not only the cross-validation strategy but also the cross-testing validation on, to the best of the authors’ knowledge, all publicly available glaucoma-labelled databases.

**Conclusions:**

These results suggest that using ImageNet-trained models is a robust alternative for automatic glaucoma screening system. All images, CNN weights and software used to fine-tune and test the five CNNs are publicly available, which could be used as a testbed for further comparisons.

## Introduction

Glaucoma is an irreversible neuro-degenerative eye disease that is considered one of the main reasons of visual disability in the world [1]. According to the World Health Organization (WHO), glaucoma affects more than 65 million people around the globe [2]. As it may be asymptomatic, early detection and treatment are important to prevent vision loss. This silent eye disease is mainly characterized by optic nerve fibre loss and that is given by the increased intraocular pressure (IOP) and/or loss of blood flow to the optic nerve. However, IOP measurement is found to be neither specific nor sensitive enough to be an effective glaucoma indicator since visual damage can be present without increased IOP.

**Fig. 1:**
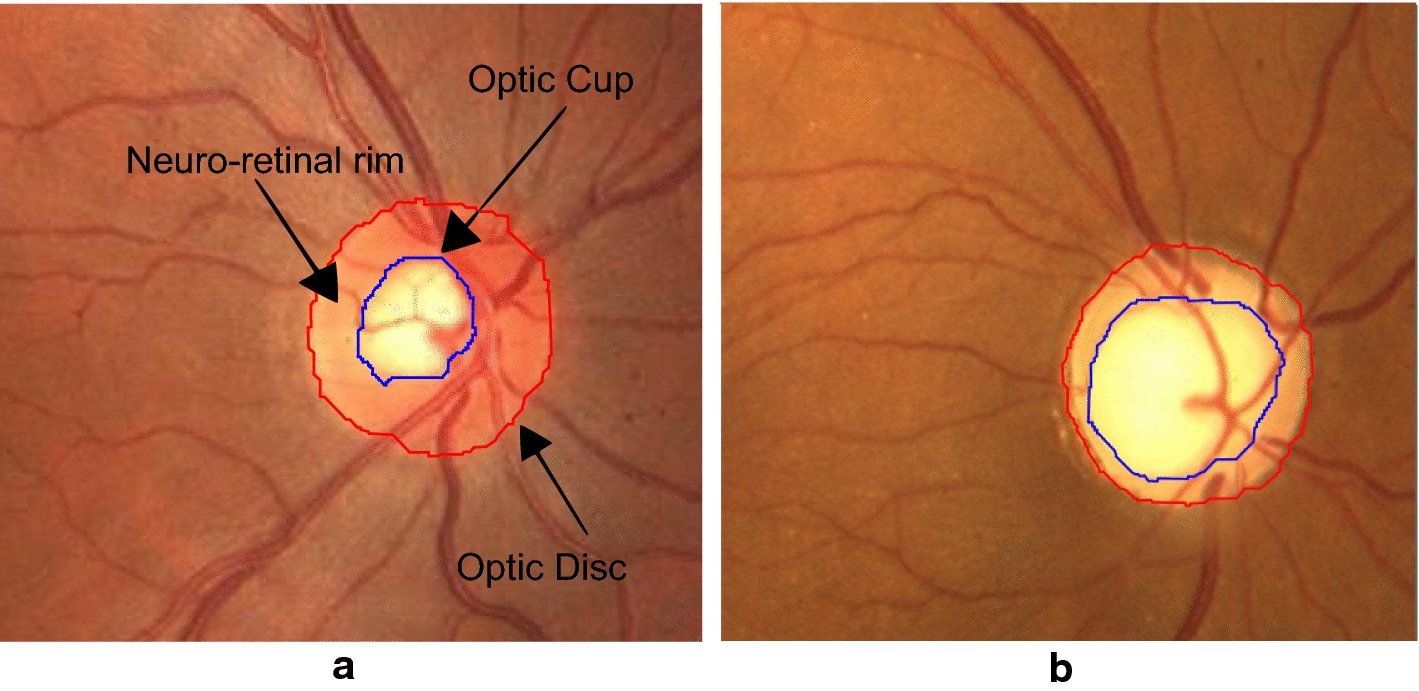
Digital fundus images cropped around optic disc. **a** Main structures of a healthy optic disc and **b** glaucomatous optic disc

The optic nerve head is where ganglion cell axons leave the eye forming the optic disc. In a fundus image, the optic disc can be visually separated into two zones, a bright and central zone called optic cup and a peripheral part called neuro-retinal rim [3]. See Fig. 1a.

While the optic disc (OD) and cup are present in all individuals, an abnormal size of the cup with respect to the optic disc is a characteristic of a glaucomatous eye, as it is shown in Fig. 1b. For that reason, different approaches have been developed towards optic cup and optic disc segmentation for glaucoma detection in colour fundus images. Some works in the literature are only focused on the optic cup and/or optic disc segmentation [4, 5] and others focus on the Cup/Disc ratio (CDR) calculation. This measurement is commonly used as a glaucoma indicator, which expresses the vertical diameter proportion of the optic disc and the cup. However, CDR measurement implies a great effort to obtain a proper optic disc and optic cup segmentation.

In this paper, five different CNN architectures are presented for glaucoma assessment. Contrary to most of the established detection techniques, this approach does not need any feature selection or accurate measurements of geometric optic nerve head structures such as CDR.

## Background

The glaucoma disease is mainly characterized by the loss of the optic nerve fibres and astrocytes. This loss can be examined by measuring the thickness of the neuro-retinal rim and the size of the optic cup with respect to the optic disc. Generally, the qualitative assessment of the optic nerve head, when using fundus images, has been the main focus of several works in the literature.

For instance, Wong et al. [6] presented a method to calculate the CDR after obtaining the optic cup and optic disc masks using level-set techniques. They tested their method on 104 images and found that their method produced results with a variation of up to 0.2 CDR units from the ground truth.

A method proposed by Joshi et al. [7] is based on anatomical evidence such as vessel bends at the cup boundary to segment the optic cup. They localised the optic cup using the vessel geometry and circular Hough transform obtaining a CDR error of $0.12 \pm 0.10$. In the study made by Yin et al. [8], they also used the knowledge-based Circular Hough Transform for segmentation of the optic disc and the optic cup. Their method was tested on 325 images obtaining an average Dice coefficient of 0.92 and 0.81, respectively.

Another approach for optic disc and optic cup segmentation is presented by Cheng et al. [9], who developed a technique to measure the CDR based on superpixel classification. They evaluated their method on 650 images achieving areas under the curve of 0.800 and 0.822 in two databases.

In the work made by Diaz-Pinto et al. [10], the authors presented an automatic algorithm to segment the optic cup and then obtain handcrafted features such as the CDR, area Cup/Disc ratio (ACDR) and the inferior–superior-nasal–temporal (ISNT) rule that checks the disc rim thickness from the fundus images. They evaluated their method on 53 images obtaining a specificity and sensitivity of 0.81 and 0.87 using the Luv colour space for optic disc and optic cup segmentation.

A work that uses other information such as the patient personal data and patient’s genome information is presented by Liu et al. [11]. They combined that information with the fundus images obtaining an area under the curve (AUC) of 0.866 for glaucoma screening, which is better than the AUC obtained when using individual personal data.

Important limitations of the methods that are based on handcrafted characteristics (CDR, Area Cup/Disc ratio (ACDR), vessel kinks and ISNT rule) is the significant disagreement in estimating them even between expert human graders. For that reason, new algorithms have been focused on automatic feature extraction such as the data-driven methods [3] and convolutional neural networks (CNNs).

In the paper published by Bock et al. [3], they proposed a data-driven method. This method is not based on accurate measurements of geometric optic nerve head structures such as the CDR. Instead, they used the idea of “Eigenimages” to extract features that are later classified by a support vector machine (SVM). They evaluated their algorithm on 575 images randomly selected from the Erlangen Glaucoma Registry (EGR), obtaining a competitive AUC of 0.88. However, the images used in their work are private and their method cannot be compared with the presented in this paper.

Convolutional neural networks (CNNs) were first introduced by Yann LeCun [12] and are biological-inspired variants of multilayer perceptrons. Since then, they have been used in computer vision and artificial intelligence. However, their relevance had not been discovered until the ImageNet competition in 2012, in which the main goal is to estimate the content of natural images for the purpose of automatic annotation using a subset of the ImageNet dataset [13]. Their success came through the use of GPUs, rectifiers such as ReLU, data augmentation techniques and new regularization techniques such as Dropout [14]. The main power of the CNN architectures relies on their ability to extract highly discriminating features at multiple levels of abstraction [15].

The first layers in a CNN extract edges at particular orientations and locations in the image. The middle layers detect structures composed of particular arrangements of edges and the last layers detect more complex structures that correspond to parts of familiar objects, or objects that are combinations of these parts.

Training a CNN from scratch is not an easy task. They require a huge amount of labelled data—a requirement that is difficult to meet in the glaucoma assessment task- and computational resources.

However, there are two alternatives to train a CNN from scratch that have been previously applied to several medical image classification tasks. The first alternative consists of fine-tuning a CNN that has been trained using a large labelled dataset from a different application (e.g., ImageNet). An example of this alternative is the work of Carneiro et al. [16], where they showed that CNN models pre-trained on natural images, such as the ImageNet, are useful in medical image applications, despite the significant differences in image appearance. The study made by Chen et al. [17] demonstrated that the use of a fine-tuned pre-trained CNN for localizing standard planes in ultrasound images outperformed the state-of-the-art for the fetal abdominal standard plane (FASP). Another example is the study made by Tajbakhsh et al. [18], in which they conducted a set of experiments for four medical imaging applications showing the use of pre-trained CNN performed as well as a CNN trained from scratch.

The second alternative consists of using an ImageNet-trained CNN as a feature extractor, where the CNN is applied to an input image and then features are extracted from a certain hidden layer of the network. Then, the extracted features are used to train a new classifier such as support vector machines (SVM), Decision Trees, K-nearest-neighbor or Naive Bayes classifier. For example, Bar et al. [19] pre-trained CNNs that were used as a feature extractor for chest pathology identification. Another study made by Razavian et al. [20] showed that using features extracted from the OverFeat network and feeding an SVM classifier, it is possible to obtain superior results compared to the highly tuned state-of-the-art systems.

For glaucoma assessment, there are also several works in the literature that employ CNNs. For instance, Chen et al. [21] proposed and trained from scratch a CNN architecture that contains six layers: four convolutional layers and two fully-connected layers; to automatically classify glaucomatous fundus images. They performed the experiments on two private databases: ORIGA-(light) which contains 650 images and SCES which contains 1676 images, achieving an AUC of 0.831 and 0.887 respectively. For ORIGA database, they trained their CNN architecture by randomly selecting 99 images, and using the remaining 551 images for test. For SCES database, they used the 650 images from ORIGA database for training, and all the 1676 images of SCES database for test. The main disadvantage is the unbalanced data. The ORIGA database is comprised of 168 glaucomatous and 482 normal fundus images and the SCES database contains 1676 fundus images of which only 46 are glaucomatous images. Another limitation of this work is that the obtained results are difficult to reproduce because the ORIGA and SCES databases are not publicly available.

A study conducted by Alghamdi et al. [22] made use of eight databases (four public and four private databases) to detect optic disc abnormality. They developed a new approach using two CNNs: one CNN was trained to first classify the optic disc region and the other CNN to classify the optic disc region into normal, suspicious and abnormal classes. However, the four public databases (DRIVE, STARE, DIARETDB1 and MESSIDOR) used in the work of Alghamdi et al. cannot be used for glaucoma classification because they were taken for different purposes. This means those images do not have any glaucoma sign or do not have glaucoma annotations. The glaucoma labelled databases they used are private and, for that reason, it is difficult to reproduce the results presented in their work.

In the study made by Abbas [23], he developed and implemented a system known as Glaucoma-Deep. This system consists of an unsupervised CNN architecture that automatically extracts features from the fundus images. Afterwards, it uses a deep-belief network (DBN) model to select the most discriminative features. In his work, Qaisar Abbas uses four databases to test his method, three of them are public and one private. Although his work shows good results (specificity: 0.9801 and sensitivity: 0.8450), details of the CNN and architecture were not given.

It is worthy to mention the work made by Orlando et al. [24], where they showed how two different CNNs, OverFeat and VGG-S, could be used as feature extractors. They also investigated how the performance of these networks behave when Contrast-limited adaptive histogram equalization (CLAHE) and vessels deletion are applied to the fundus images. In their work, they used Drishti-GS1 database to test the performance of the fine-tuned CNNs. They observed that OverFeat CNN performed better than VGG-S, obtaining an AUC of 0.7626 and 0.7180, respectively. The main limitation of this work is the small number of images (101 images) used to test the performance of the CNNs. However, their method achieved a competitive AUC score with respect to other existing strategies.

In this paper, an analysis of five different ImageNet-trained CNN architectures used as glaucoma classifier is presented. They were fine-tuned and tested using exclusively public databases, which differ to most of the presented works in the literature that use private databases. The high accuracy, specificity and sensitivity obtained from this analysis suggest that ImageNet-trained CNN architectures are a robust alternative for an automatic glaucoma detection algorithm. These CNNs work properly in colour fundus images belonging to five different public databases (1707 images) with high variability grade. Furthermore, we introduce of a new public database, ACRIMA, composed of 705 labelled images (396 glaucomatous and 309 normal images), that could be used as a testbed for further comparisons between methods developed for glaucoma classification.

## Material and methods

### ACRIMA: a new public database

There are few publicly available databases with glaucoma-labelled images that can be used for the evaluation of glaucoma classification methods. For that reason, the authors are pleased to introduce a new public available glaucoma-labelled database called ACRIMA.^1^ The images of this database come from the ACRIMA project (TIN2013-46751-R) founded by the Ministerio de Economía y Competitividad of Spain, which aims to the development of automatic algorithms for retinal disease assessment.

#### Database description

ACRIMA database is composed of 705 fundus images (396 glaucomatous and 309 normal images). They are part of the ACRIMA project and were obtained from glaucomatous and normal patients with their previous consent and in accordance with the ethical standards laid down in the 1964 Declaration of Helsinki. All patients were selected by experts based on their criteria and clinical findings during the examination. Most of the fundus images from this database were taken from the left and right eye previously dilated and centred in the optic disc. Some of them were discarded because of artefacts, noise and poor contrast. They were captured using the Topcon TRC retinal camera and IMAGEnet^®^ capture System. Images were taken with a field of view of 35°.

**Fig. 2:**
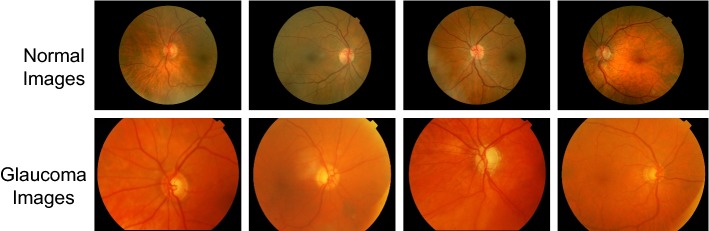
Examples of new publicly available database. Normal and Glaucoma fundus images from the new publicly available database (ACRIMA)

All images from ACRIMA database were annotated by two glaucoma experts with 8 years of experience. No other clinical information was taken into account while providing labels for the images. This first version of ACRIMA database could only be used for classification tasks. Optic disc and optic cup segmentation are not provided. Examples of images from the ACRIMA database are shown in Fig. 2.

### Other databases

Apart from the ACRIMA database, other four public databases were used in this work: HRF database [25], which contains 45 images; Drishti-GS1 database [4], which consists of 101 images; RIM-ONE database [26], which is composed of 455 images; and sjchoi86-HRF [27] database which is composed of 401 images. All these databases are shown in detail in Table 1.

**Table 1:** List of all the public available databases with glaucoma labels

Database	Glaucoma	Normal	Total
HRF [25]	27	18	45
Drishti-GS1 [4]	70	31	101
RIM-ONE [26]	194	261	455
sjchoi86-HRF [23, 27]	101	300	401
*ACRIMA*	*396*	*309*	*705*
	788	919	1707

Italic represents the new publicly available database

For all the experiments executed in this work, the open source Deep Learning library Keras [28] and NVIDIA Titan V GPU were used. Keras library is a simple way to use, implement and fine-tune CNNs architectures built on top of Theano, TensorFlow or CNTK.

### Preprocessing

The fundus images used for the fine-tuning process were automatically cropped around the optic disc using a bounding box of 1.5 times the optic disc radius. Except the RIM-ONE database which came originally cropped around the optic disc. To do this cropping, we employed the method proposed in [29]. In their method, Xu et al. used a basic CNN to find the most probable pixels in the optic disc region. Then, they sort out those candidate pixels via using a threshold.

**Fig. 3:**
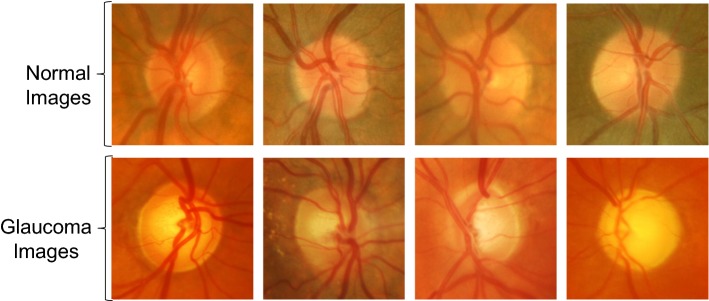
Examples of the cropped fundus images. Cropped fundus images used for fine-tuning and testing the CNNs

Cropping the images around the optic disc has a clinical reason, glaucoma disease affects mainly the optic disc and its surroundings. Moreover, it was shown by Orlando et al. [24] that cropping the images around the optic disc turned out to be a more efficient way than using the whole images when using CNN for glaucoma assessment. Examples of the images used for fine-tuning the CNNs are shown in Fig. 3.

### ImageNet-trained CNN architectures

In this work, the VGG16 [15], VGG19 [15], InceptionV3 [30, 31], ResNet50 [32] and the Xception [33] architectures were fine-tuned to the glaucoma assessment task using their ImageNet-trained versions available in the Keras core. To use these networks to this task, the last fully connected layer of each CNN was changed for a global average pooling layer (GlobalAveragePooling2D) followed by a fully connected layer of two nodes representing two classes (glaucoma and healthy) and a softmax classifier. Therefore, counting the new top layers on each CNN, the total number of Keras layer in the VGG16 and VGG19 network architectures were 20 and 23, respectively. The InceptionV3 architecture is composed by 312 Keras layers and the ResNet50 and Xception architecture are composed by 176 and 133 Keras layers, respectively. Note that to fine-tune the models, images were automatically cropped around the optic disc as it was previously mentioned.

In order to obtain the best performance of each model, we carried out several experiments varying the number of fine-tuned layers and the number of epochs. First, for the number of fine-tuned layers, we started by fine-tuning the last weighted layer of the CNN architectures, keeping the other layers in a “not-trainable” mode. Afterwards, the number of fine-tuned layers was increased until updating all the layers in the CNN.

The second experiment consisted in analysing the impact of the number of epochs that present the best performance of each architecture. Other hyper-parameters such as batch size, learning rate, etc were fixed while varying the number of fine-tuned layers and number of epochs. For instance, we set the number of epochs in 100, for the first experiment, and the number of layers in “not-trainable” mode was set in 0 for the second experiment. For both experiments, the Stochastic Gradient Descent (SGD) was used as the optimizer, the batch size was set to 8, the learning rate to $1{e^{-4}}$ and the momentum to 0.9. All these hyper-parameters were optimally chosen to get the best performance in our set of experiments.

Additionally to these experiments, we also evaluated the performance of the CNNs (VGG16, VGG19, Inceptionv3, ResNet50 and Xception) using the k-fold Cross-Validation technique with $k=10$, following the procedure described in [34]. In order to avoid overfitting and increase the robustness of the models, available images were augmented by using random rotations, zooming by a range between 0 and 0.2 and horizontal and vertical flipping. The images were also resized to the default input size of each CNN architecture ($224\times 224$ for VGG16, VGG19 and ResNet50 and $299\times 299$ for Inceptionv3 and Xception).

A particular performance evaluation of the CNNs, using datasets that were not used during the training stage, was also carried out. Different to most of the works in the literature, this experiment checks the performance of the CNNs on complete databases that the system have not seen during the training stage.

The final experiment is the comparison between the best of the five previously mentioned CNNs with a state-of-the-art algorithm that also uses public databases.

## Experimental results and discussion

**Fig. 4:**
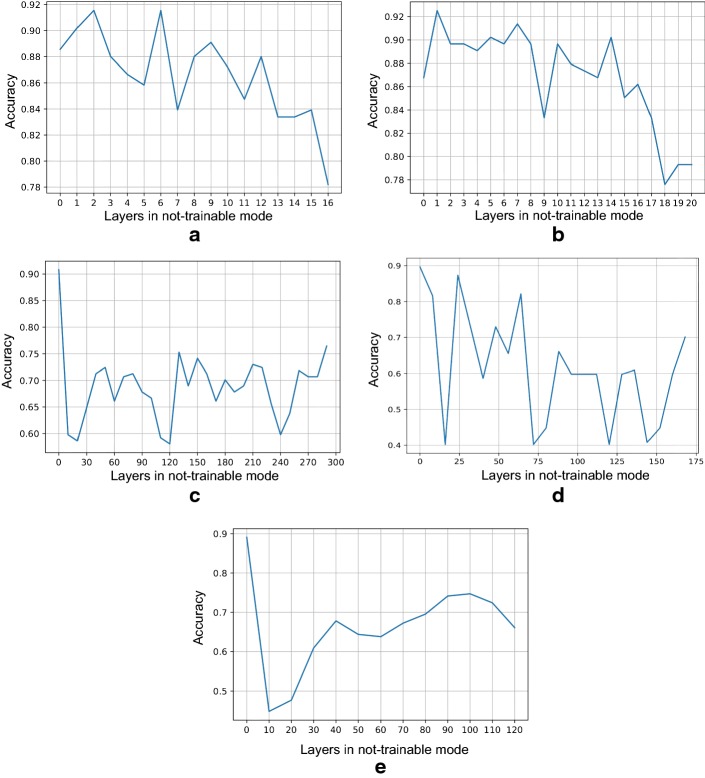
Plot showing the effect of fine-tuning different number of layers. **a** VGG16, **b** VGG19, **c** InceptionV3, **d** ResNet50 and **e** Xception architecture

As mentioned in the previous section, two initial experiments to see the effect of fine-tuning different number of layers and the number of epochs were carried out. In Fig. 4, it is possible to see the trend in improvement from shallow tuning to deep tuning for the glaucoma assessment task of each CNN. The x-axis represents the number of layers in “not-trainable” mode of each CNN. The experiment starts from fine-tuning all layers (x-axis = 0) until fine-tuning only the last trainable layer in the model.

From this experiment, we can see that fine-tuning all the trainable layers in the CNN, or doing deep tuning, is the best option when trying to obtain the best performance, as it was also demonstrated by Tajbakhsh et al. [18] in their paper.

**Fig. 5:**
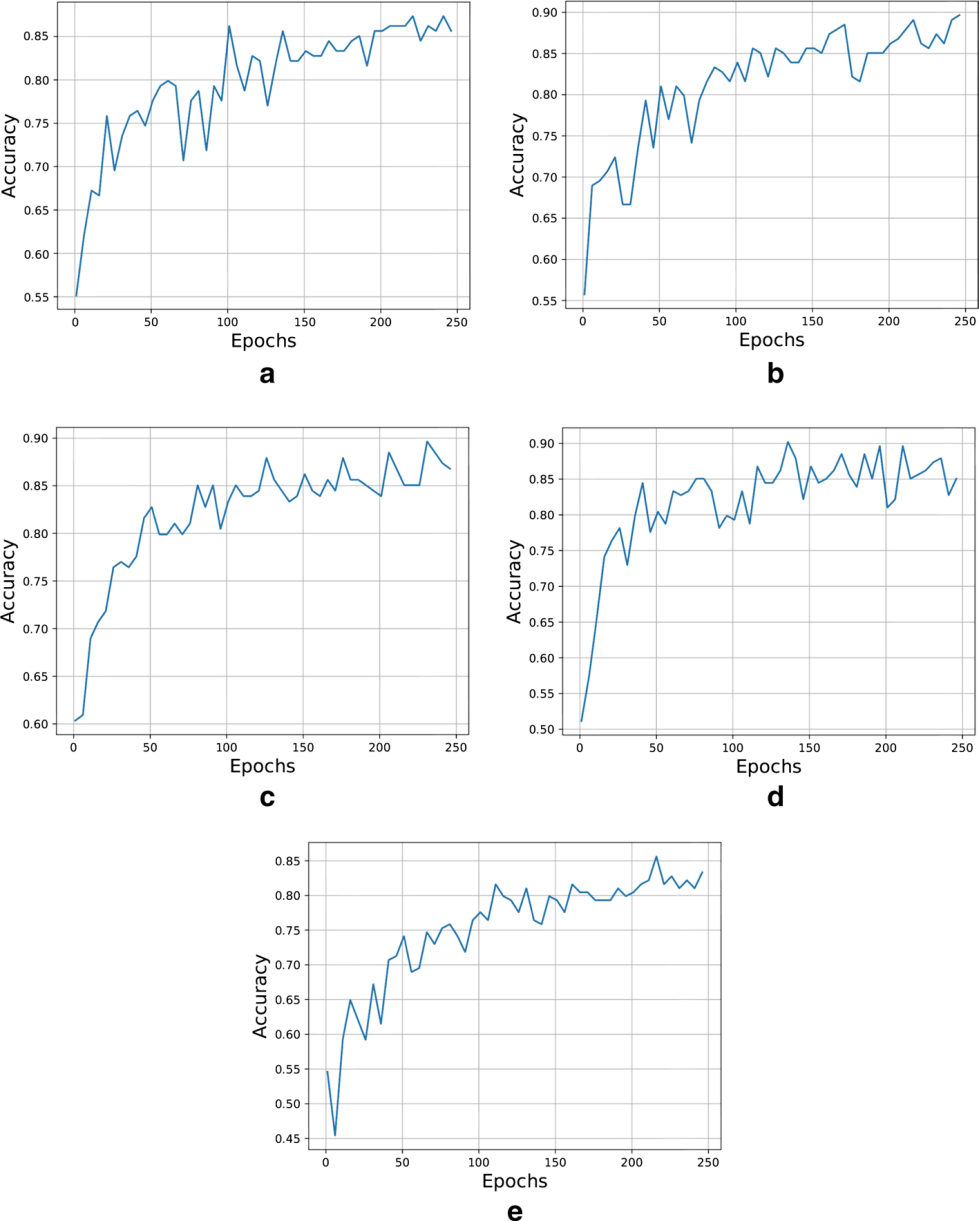
Plot showing the effect of number of epochs. **a** VGG16, **b** VGG19, **c** InceptionV3, **d** ResNet50 and **e** Xception architecture

For the other initial experiment, we put in trainable mode all layers and see the performance of the CNN when fine-tuning from 1 to 250 epochs. It is important to highlight that the performance evaluation for this experiment was carried out on the validation set. The result of this experiment can be seen in Fig. 5. From this experiment, we could see that around 200 is the optimum number of epochs to get the best performance for the fine-tuning process in our set of experiments.

For model evaluation, 10-fold cross-validation was performed. Therefore, 10 values of area under the curve (AUC), accuracy, specificity, sensitivity and F-score were obtained. Afterwards, the average and standard deviation of these values were calculated for each CNN architecture. Results for each fine-tuned model are presented in Table 2. Additional to the area under the curve (AUC), accuracy, specificity, sensitivity and F-score, we also calculated for each fold the P-value by using the Mann–Whitney U test [35, 36] and used the bootstrapping technique to calculate the confidence interval for the AUC values. The P-value was calculated by comparing the ground-truth distribution and labels distribution obtained from each model. The label distribution of each model was obtained by thresholding the obtained probabilities from each CNN (glaucoma if score > 0.5, normal otherwise). The idea of providing confidence intervals for each CNN is to provide a likelihood that the AUC of each model will fall between the range when making predictions on new data. To calculate these ranges, we used the trained models in each fold to predict random selected images from the test set. Ten bootstrap repetitions for each fold were performed, meaning 100 repetitions for each model.

**Table 2:** Results for each model doing deep tuning and 10-fold cross validation

Model name	AUC	95% confidence interval	Accuracy	Specificity	Sensitivity	Fscore	P-value
VGG16 [15]	0.9632 (0.0149)	95.81–96.87%	0.8948 (0.0253)	0.8816 (0.0612)	0.9057 (0.0331)	0.9005 (0.0231)	0.3240
VGG19 [15]	0.9686 (0.0158)	96.45–97.39%	0.9069 (0.0318)	0.8846 (0.0362)	0.9240 (0.0434)	0.9125 (0.0312)	0.3607
InceptionV3 [30]	0.9653 (0.0135)	96.12–97.32%	0.9000 (0.0201)	0.8752 (0.0358)	0.9216 (0.0311)	0.9056 (0.0236)	0.3126
ResNet50 [32]	0.9614 (0.0171)	95.62–96.77%	0.9029 (0.0249)	0.8943 (0.0350)	0.9105 (0.0282)	0.9076 (0.0251)	0.3885
Xception [33]	0.9605 (0.0170)	95.92–97.07%	0.8977 (0.0264)	0.8580 (0.0398)	0.9346 (0.0247)	0.9051 (0.0274)	0.2729

**Fig. 6:**
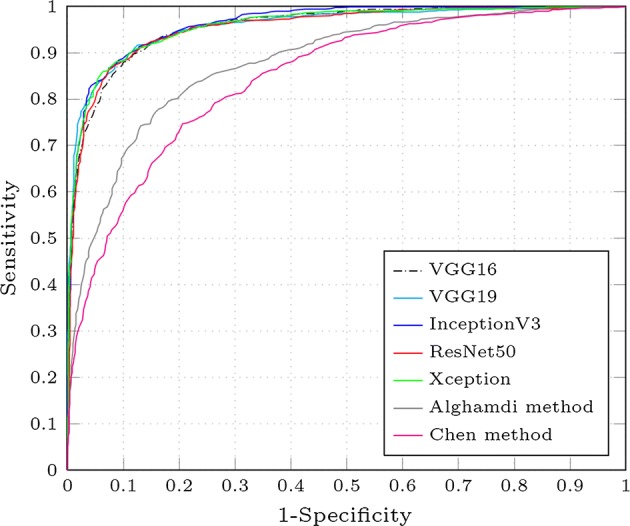
Average ROC curves for each CNN. Average ROC curves for each fine-tuned CNN architecture (VGG16 [15], VGG19 [15], InceptionV3 [31], ResNet50 [32] and Xception [33]), the Alghamdi [22] and Chen method [21]

Additionally to the measurements reported in Table 2, the ROC curves for the average specificity and sensitivity, obtained by performing 10-folds cross-validation, were plotted in Fig. 6. It is possible to see that all the proposed fine-tuned models have a really good performance for the glaucoma assessment task. As the performance of the models is comparable, characteristics of the CNN such as the number of parameters can be used to determine what model is better than the others.

In order to compare the obtained results with other works in the literature, we implemented, trained and tested on the same images the neural networks proposed by Chen et al. [21] and Alghamdi et al. [22]. The obtained results from these models are presented in Fig. 6. Although they obtained a high area under the ROC curve using their methods, the systems proposed in this paper clearly outperform them.

In Table 3, the number of parameters and the obtained AUC of each CNN architecture are shown. It is possible to see that, although VGG16 and VGG19 present higher AUC than the Xception architecture, they have much more parameters to fine-tune, which requires more computation power and resources. Therefore, the Xception architecture presents a better trade-off between the number of parameters and obtained AUC than the other architectures.

**Table 3:** Number of parameters and obtained AUC for each architecture

Model name	# parameters (in millions)	AUC
VGG16	138	0.9632 (0.0149)
VGG19	144	0.9686 (0.0158)
InceptionV3	23	0.9653 (0.0135)
ResNet50	25	0.9614 (0.0171)
*Xception*	*22*	*0.9605 (0.0170)*

The best architecture in terms of AUC and number of parameters (in italic)

**Fig. 7:**
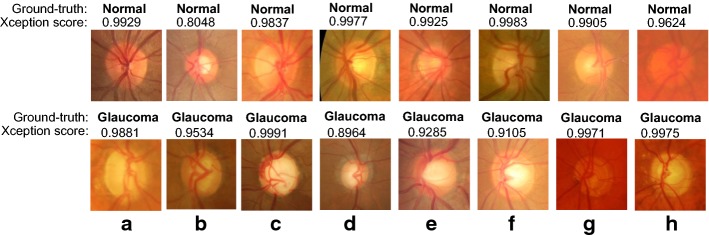
Examples of correct normal and glaucoma classification. Samples of well-classified normal and glaucoma images by the Xception architecture

**Fig. 8:**
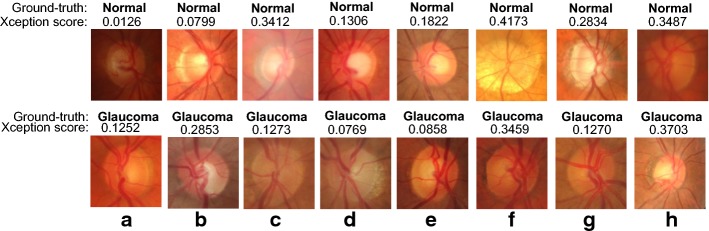
Examples of miss-classified normal and glaucoma images. Samples of normal and glaucoma images miss-classified by the Xception architecture

In Figs. 7 and 8, samples of the classification results obtained from the Xception architecture are presented, including correct and incorrect classification examples. Low score values (close to 0) mean miss-classification and score values close to 1 mean correct classification.

We believe a possible reason the CNN miss-classified glaucomatous images is because they do not have the big bright area inside the optic disc. As previously mentioned, the optic nerve loss is, most of the times, visible from the fundus image. The brighter area (or optic cup) inside the optic disc is usually bigger in a glaucomatous image. It seems the CNN learned to recognise glaucomatous images based on this criteria. Images with no big bright area are classified as normal (see lower row images in Fig. 8). Other possible factors for the miss-classification are the low quality of the images (see upper row Fig. 8a, h).

**Fig. 9:**
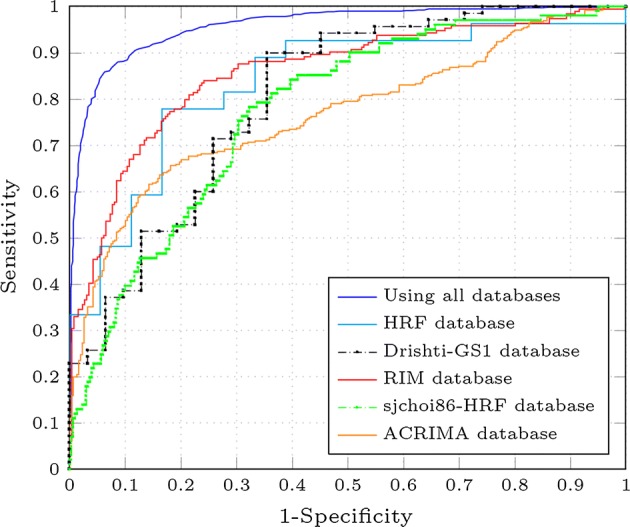
ROC curves for the Xception architecture in different experiments. ROC curves for the Xception architecture in different experiments. When using HRF, Drishti-GS1, RIM, sjchoi86-HRF and ACRIMA database only as test set and using all the databases

Given the changes of illumination, the glaucoma assessment using fundus images is not an easy task. A developed method that properly classifies images from a certain database/s does not necessarily perform well when it is applied to images from a different database. A critical experiment that evaluates the performance of a glaucoma classifier consists of using images that come from a different sensor or database. For that reason, five different experiments using the Xception architecture and all public glaucoma-labelled databases (HRF, Drishti-GS1, RIM, sjchoi86-HRF and ACRIMA) were done. First, the Xception architecture was fine-tuned using all the databases except the images that belong to the database to be tested. Secondly, the trained model is tested on the desired database. This approach is repeated to test HRF, Drishti-GS1, RIM, sjchoi86-HRF and ACRIMA database. The results obtained from these experiments are presented in Fig. 9 and Table 4, in which is possible to see that although the Xception architecture was fine-tuned without using images from these databases, its performance is promising.

**Table 4:** Obtained results for HRF, Drishti-GS1, RIM, sjchoi86-HRF and the new public database ACRIMA, using Xception architecture represented in AUC, AUC’s confidence interval, Accuracy, sensitivity and specificity

Database	AUC	AUC’s 95% confidence interval	Accuracy	Sensitivity	Specificity	# images
HRF	0.8354	50.00–100.00%	0.8000	0.8333	0.7778	45
Drishti-GS1	0.8041	50.49–92.55%	0.7525	0.7419	0.7143	101
RIM-ONE	0.8575	77.53–91.12%	0.7121	0.7931	0.7990	455
sjchoi86-HRF	0.7739	66.44–86.85%	0.7082	0.7033	0.7030	401
ACRIMA	0.7678	68.41–81.81%	0.7021	0.6893	0.7020	705

**Fig. 10:**
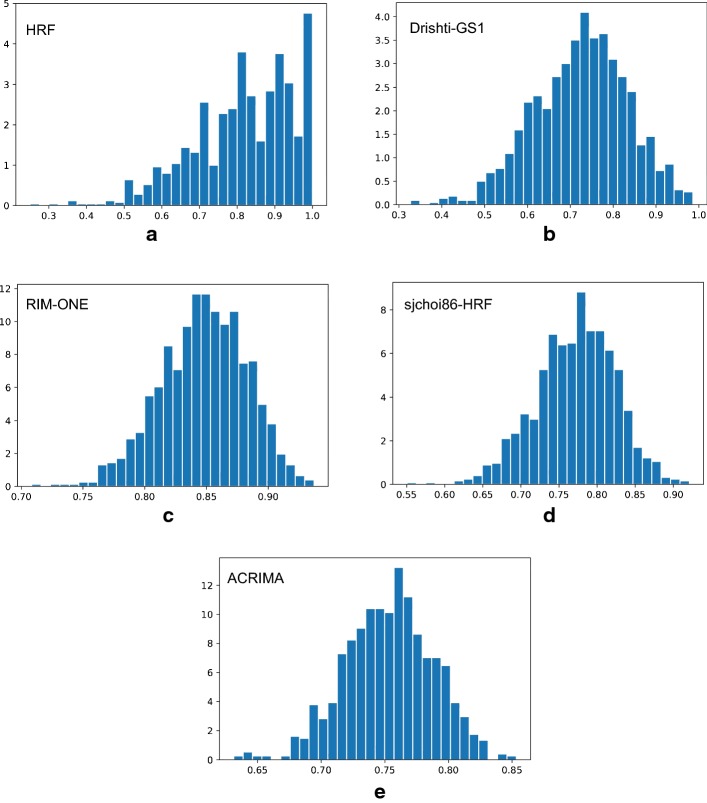
Distribution of the AUC values per database. Histogram of the 1000 AUC values when evaluating the Xception architecture using the bootstrapping technique. It shows the results of the bootstrap repetitions when using **a** HRF, **b** Drishti-GS1, **c** RIM-ONE, **d** sjchoi86-HRF and **e** ACRIMA as test only

The AUCs confidence intervals presented in Table 4 were calculated from 1000 bootstrap replications. We used the trained model of each experiment and randomly selected images from the test set to obtain an AUC value for each bootstrap replication. In Fig. 10 is possible to see the distribution of the 1000 AUC values.

It can be seen from Table 4 how the CNN accuracy drops (about 15%) when predicting on images from different databases than what were used for training. This drop in accuracy shows the CNN does not generalize well when classifying images from different databases. This is because images from different databases are labeled in two different ways: the first is when experts rely on the patient’s medical history and the fundus image itself to assign a label and the other way is when experts rely only on the visual information in the fundus image. The last way of labelling the images increase the noise in labels and makes even more difficult the generalization of an automatic glaucoma classification system. Taking into account that the CNN is only based on the raw pixel information to classify the images, it is expected that the accuracy is dramatically affected when testing the system on images from different databases. Different databases mean different labelling systems.

Analysing in depth the results from Table 4, two main results can be stood out. The first relies on checking the real performance of the CNNs when the model is tested on images from databases that were not used during the training stage. It is not only unseen data but whole databases with different expert labelers and image characteristics. In contrast to most of the works in the literature, in which they test their systems on unseen data but using images from the same database with a similar appearance.

The second result is to show the difficulty of this problem. The scarcity of labelled images, and the fact that the available images come from different databases, makes the development of a robust glaucoma classifier that works for every database a complex problem.

A possible solution to increase the performance of the CNNs is by using part of the data for training and part for test. In this way, we could test the CNNs on unseen data but using images from databases used during the training stage. Another solution could be to retrain the CNNs when trying to classify images from databases different to the ones used for training.

We proved this drop in accuracy does not occur when training the CNN on 70% of the data (1195 images) and testing on 30% of the data (512 images) from the same databases. From this experiment, we obtained an AUC of 0.9464, an accuracy of 0.8908, a sensitivity of 0.9175 and a specificity of 0.8571. It shows that the CNN performs well when classifying unseen images from the same databases that were used for training.

Thanks to the public availability of the Drishti-GS1 database, a comparison with other state-of-the-art algorithms that used this database is also possible. For instance, in the work developed by Chakravarty et al. [37], they obtained an AUC of 0.78 when tested their method on this database. Another example is the work presented by Orlando et al. [24], in which they obtained an AUC of 0.76 using pre-trained CNNs applied to the Drishti-GS1 database. It can be seen in Table 4 that the method proposed in this paper outperforms (AUC = 0.8041) the existing works. Moreover, it must be taken into account that Chakravarty et al. [37] and Orlando et al. [24] evaluated their methods using the same database for training and test unlike the experiments performed in this work where Drishti-GS1 database is only used for test. This could be the reason for the low gain of our method with respect to the others. This gain significantly increases (AUC = 0.9605 for Xception. See Table 3) when Drishti-GS1 database is used for both stages, training and test.

**Fig. 11:**
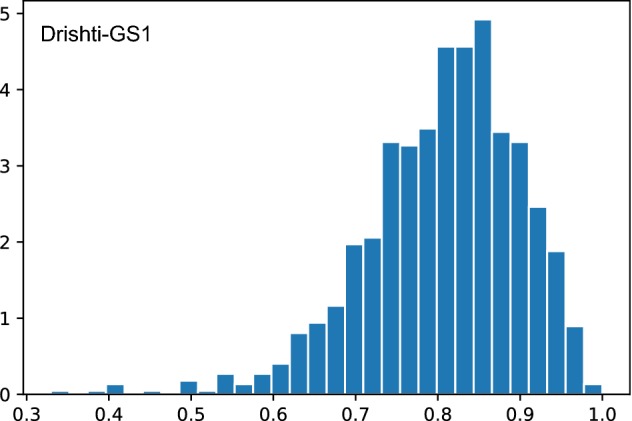
Distribution of the AUC values for the Drishti-GS1 database. Histogram of the 1000 AUC values obtained from the bootstrapping technique evaluating the Xception architecture trained and tested on the Drishti-GS1 database

An additional experiment was performed using the Xception architecture and only the Drishti-GS1 database. In order to do a fair comparison with the method published in [24], the images in this database were randomly divided into training, validation and test as it was done in their work: 70% for training, 30% for test and 30% of the training images were selected for the validation set. For this experiment, the batch size was decreased to 1 and the learning rate to $1e^{-3}$ because of the small number of images in the Drishti-GS1 database (101 images in total). With this configuration, an AUC of 0.8261 with a 95% confidence interval of 59.71–95.20% was obtained. Additionally, we used the bootstrapping technique and the trained model to obtain 1000 AUC values and check its distribution (see Fig. 11).

The previous experiments, the analysis of those CNNs and the obtained results prove to be an important advance to the glaucoma classification task using fundus images.

### Computation time

We used the Keras library and a Titan Xp GPU for this work. We measured the time consumed for each model with the configuration previously described (regularization technique, number of fine-tuned layers, number of images for training, batch size, etc). The average time to fine-tune is 1 h and 40 min for the VGG16, 1 h and 55 min for the VGG19, 1 h and 45 min for the InceptionV3, 1 h and 15 min for the ResNet50 and 2 h and 40 min for the Xception architecture. The time for each architecture was obtained by averaging the time consumed for each fold during the fine-tuning stage. Once the models were fine-tuned, 46 ms are needed to assign a glaucoma probability for each retinal image.

## Conclusion

In this work, five different ImageNet-trained CNN architectures (VGG16, VGG19, InceptionV3, ResNet50 and Xception) were analysed and used as glaucoma classifiers. Using only publicly available databases, the Xception architecture shows the best performance for glaucoma classification, which was evaluated as the trade-off between the AUC and the number of parameters of the CNN. Based on the 1707 images and data augmentation technique, an average AUC of *0.9605* with a 95% confidence interval of 95.92–97.07%, an average specificity of *0.8580* and an average sensitivity of *0.9346* were obtained after fine-tuned the Xception architecture, significantly improving other state-of-the-art works.

Moreover, an additional analysis shows that the fine-tuned model has competitive performance when it is tested on images that come from a completely different database. This experiment differs to the common approach in which a subset of a database is used for training and the other subset is used for testing. Using the ACRIMA database as test set only, an AUC of 0.7678 with a 95% confidence interval of 68.41–81.81% was obtained. The same experiment was done for the other four public databases: HRF, Drishti-GS1, RIM-ONE, sjchoi86-HRF, obtaining an AUC of 0.8354, 0.8041, 0.8575 and 0.7739, respectively.

ACRIMA^2^ database is composed of 396 glaucomatous images and 309 normal images and could be easily used as a testbed for further comparisons and/or analysis. The authors encourage the scientist community to test their models using the new publicly available database and compare their results with the method proposed in this paper.

As a further work, the use of synthetic images for training the CNNs could be of great interest to increase the number of training images. In this way, we could train even more robust glaucoma classifiers.

## Limitations of the study

Although we used a rather big database and used data augmentation to fine-tune the CNNs, there is still a limitation when trying to generalise. As it was shown in Table 4, the performance decreased when testing the CNN on databases different from those used for training. Additionally to this problem, we found that the different labelling criteria is another issue we faced when developing automatic glaucoma assessment systems. Most of the publicly available databases differ in the way they are labelled, the information clinical experts used to assess images and the quality of the fundus images.

## Footnotes

1 Link to ACRIMA database. It will be publicly available after the paper is accepted.

2 Link to the ACRIMA database during review process.

## References

[1] World Health Organization. Bulletin of the World Health Organization, Vol 82(11). 2004. http://www.who.int/bulletin/volumes/82/11/en/infocus.pdf?ua=1. Accessed 5 May 2016.

[2] Bourne RRA (2006). Worldwide glaucoma through the looking glass. Br J Ophthalmol.

[3] Bock R, Meier J, Nyúl LG, Hornegger J, Michelson G (2010). Glaucoma risk index: automated glaucoma detection from color fundus images. Med Image Anal.

[4] Sivaswamy J, Krishnadas SR, Joshi GD, Jain M, Ujjwal A, ST Drishti. Retinal image dataset for optic nerve head (ONH) segmentation. In: 2014 IEEE 11th international symposium on biomedical imaging (ISBI). 2014, p. 53–6. 10.1109/ISBI.2014.6867807.

[5] Morales S, Naranjo V, Angulo J, Alcañiz M (2013). Automatic detection of optic disc based on PCA and mathematical morphology. IEEE Trans Med Imag.

[6] Wong DWK, Liu J, Lim JH, Jia X, Yin F, Li H, Wong TY. Level-set based automatic cup-to-disc ratio determination using retinal fundus images in ARGALI. In: 30th Annual International IEEE EMBS Conference, vol. 30. 2008, p. 2266–9. 10.1109/IEMBS.2008.4649648. 10.1109/IEMBS.2008.4649648 19163151

[7] Joshi GD, Sivaswamy J, Krishnadas SR (2011). Optic disk and cup segmentation from monocular color retinal images for glaucoma assessment. IEEE Trans Med Imag.

[8] Yin F, Liu J, Wong DWK, Tan NM, Cheung C, Baskaran M, Aung T, Wong TY. Automated segmentation of optic disc and optic cup in fundus images for glaucoma diagnosis. In: 2012 25th IEEE international symposium on computer-based medical systems (CBMS). 2012, p. 1–6. 10.1109/CBMS.2012.6266344.

[9] Cheng J, Liu J, Xu Y, Yin F, Wong DWK, Tan N-M, Tao D, Cheng C-Y, Aung T, Wong TY. Superpixel classification based optic disc and optic cup segmentation for glaucoma screening. In: IEEE transactions on medical imaging, vol. 32. 2013, p. 1019–32. 10.1109/TMI.2013.2247770. 10.1109/TMI.2013.2247770 23434609

[10] Diaz-Pinto A, Morales S, Naranjo V, Alcocer P, Lanzagorta A. Glaucoma diagnosis by means of optic cup feature analysis in color fundus images. In: 24th European signal processing conference (EUSIPCO), vol. 24. 2016, p. 2055–9. 10.1109/EUSIPCO.2016.7760610.

[11] Liu J, Zhang Z, Wong DWK, Xu Y, Yin F, Cheng J, Tan NM, Kwoh CK, Xu D, Tham YC, Aung T, Wong TY (2013). Automatic glaucoma diagnosis through medical imaging informatics. J Am Med Inf Assoc.

[12] LeCun Y (1989). Generalization and network design strategies.

[13] Russakovsky O, Deng J, Su H, Krause J, Satheesh S, Ma S, Huang Z, Karpathy A, Khosla A, Bernstein M, Berg AC, Fei-Fei L (2015). ImageNet large scale visual recognition challenge. Int J Comput Vis.

[14] Srivastava N, Hinton G, Krizhevsky A, Sutskever I, Salakhutdinov R (2014). Dropout: a simple way to prevent neural networks from overfitting. J Mach Learn Res.

[15] Simonyan K, Zisserman A. Very deep convolutional networks for large-scale image recognition. 2014. ArXiv e-prints arxiv:abs/1409.1556.

[16] Carneiro G, Nascimento J, Bradley AP. In: Navab N, Hornegger J, Wells WM, Frangi AF, eds. Unregistered multiview mammogram analysis with pre-trained deep learning models. 2015, p. 652–60. Cham: Springer. 10.1007/978-3-319-24574-4_78.

[17] Chen H, Ni D, Qin J, Li S, Yang X, Wang T, Heng PA (2015). Standard plane localization in fetal ultrasound via domain transferred deep neural networks. IEEE J Biomed Health Inf.

[18] Tajbakhsh N, Shin JY, Gurudu SR, Hurst RT, Kendall CB, Gotway MB, Liang J (2016). Convolutional neural networks for medical image analysis: full training or fine tuning?. IEEE Trans Med Imag.

[19] Yaniv B, Idit D, Lior W, Hayit G. Deep learning with non-medical training used for chest pathology identification. In: Proceedings of SPIE 9414, medical imaging 2015: computer-aided diagnosis. 2015, p. 94140–7. 10.1117/12.2083124.

[20] Razavian AS, Azizpour H, Sullivan J, Carlsson S. CNN features off-the-shelf: an astounding baseline for recognition. 2014. arXiv e-prints arxiv:abs/1403.6382.

[21] Chen X, Xu Y, Wong DWK, Wong TY, Liu J. Glaucoma detection based on deep convolutional neural network. In: 2015 37th annual international conference of the IEEE engineering in medicine and biology society (EMBC). 2015, p. 715–8. 10.1109/EMBC.2015.7318462. 10.1109/EMBC.2015.7318462 26736362

[22] Alghamdi HS, Tang HL, A.Waheeb S, Peto T. Automatic optic disc abnormality detection in fundus images: a deep learning approach. In: OMIA3 (MICCAI 2016). 2016, p. 17–24. 10.17077/omia.1042.

[23] Abbas Q (2017). Glaucoma-deep: detection of glaucoma eye disease on retinal fundus images using deep learning. Int J Adv Comput Sci Appl.

[24] Orlando JI, Prokofyeva E, del Fresno M, Blaschko MB. Convolutional neural network transfer for automated glaucoma identification. In: SPIE proceedings. 2017, p. 10160–10. 10.1117/12.2255740.

[25] Budai A, Bock R, Maier A, Hornegger J, Michelson G (2013). Robust vessel segmentation in fundus images. Int J Biomed Imag.

[26] Fumero F, Alayon S, Sanchez JL, Sigut J, Gonzalez-Hernandez M. RIM-ONE: an open retinal image database for optic nerve evaluation. In: 2011 24th international symposium on computer-based medical systems (CBMS). 2011, p. 1–6. 10.1109/CBMS.2011.5999143.

[27] sjchoi86: sjchoi86-HRF Database. GitHub. 2017. Accessed 2 Feb 2017.

[28] Chollet F, et al. Keras. GitHub. 2015. Accessed 21 Feb 2017.

[29] Xu P, Wan C, Cheng J, Niu D, Liu J (2017). Optic disc detection via deep learning in fundus images. Fetal, infant and ophthalmic medical image analysis.

[30] Szegedy C, Liu W, Jia Y, Sermanet P, Reed S, Anguelov D, Erhan D, Vanhoucke V, Rabinovich A. Going deeper with convolutions. In: 2015 IEEE conference on computer vision and pattern recognition (CVPR). 2015, p. 1–9. 10.1109/CVPR.2015.7298594.

[31] Szegedy C, Vanhoucke V, Ioffe S, Shlens J, Wojna Z. Rethinking the inception architecture for computer vision. In: The IEEE conference on computer vision and pattern recognition (CVPR). 2016, p. 2818–26.

[32] He K, Zhang X, Ren S, Sun J Deep residual learning for image recognition. In: The IEEE conference on computer vision and pattern recognition (CVPR). 2016.

[33] Chollet F. Xception: Deep learning with depthwise separable convolutions. ArXiv e-prints. 2016. arxiv:abs/1610.02357.

[34] Hastie T, Tibshirani R, Friedman J (2009). The elements of statistical learning.

[35] Mason SJ, Graham NE (2002). Areas beneath the relative operating characteristics (roc) and relative operating levels (rol) curves: statistical significance and interpretation. Q J R Meteorol Soc.

[36] Mann HB, Whitney DR (1947). On a test of whether one of two random variables is stochastically larger than the other. Ann Math Statist.

[37] Chakravarty A, Sivaswamy J Glaucoma classification with a fusion of segmentation and image-based features. In: 2016 IEEE 13th international symposium on biomedical imaging (ISBI). 2016, p. 689–92. 10.1109/ISBI.2016.7493360.

